# Channel Selection and Feature Projection for Cognitive Load Estimation Using Ambulatory EEG

**DOI:** 10.1155/2007/74895

**Published:** 2007-08-21

**Authors:** Tian Lan, Deniz Erdogmus, Andre Adami, Santosh Mathan, Misha Pavel

**Affiliations:** ^1^Department of Biomedical Engineering, Oregon Health and ScienceUniversity, Portland, OR 97239, USA; ^2^Department of Computer Science, University of Caxias do Sul, 95070-560 Caxias do Sul, RS, Brazil; ^3^Human Centered Systems, Honeywell Laboratories, Minneapolis, MN 55401, USA

## Abstract

We present an ambulatory cognitive state classification system to assess the subject's mental load based on EEG measurements. The ambulatory cognitive state estimator is utilized in the context of a real-time augmented cognition (AugCog) system that aims to enhance the cognitive performance of a human user through computer-mediated assistance based on assessments of cognitive states using physiological signals including, but not limited to, EEG. This paper focuses particularly on the offline channel selection and feature projection phases of the design and aims to present mutual-information-based techniques that use a simple sample estimator for this quantity. Analyses conducted on data collected from 3 subjects performing 2 tasks (n-back/Larson) at 2 difficulty levels (low/high) demonstrate that the proposed mutual-information-based dimensionality reduction scheme can achieve up to 94% cognitive load estimation accuracy.

## 1. INTRODUCTION

Following the successful demonstration of a P300 oddball detector [1], many brain computer interfaces (BCIs) are designed on similar concepts [2] —evoked response potential (ERP) detection or sliding window classification. Artifact removal using adaptive filtering source separation techniques have been proposed [3, 4], wavelet coefficients [5], short-term power spectrum [6–8], and chaos/fractal structure [9, 10] have been investigated as potential features. Various standard classifiers including linear discriminants, neural networks, and support vector machines are employed [11–16], parametric and nonparametric approximate Bayes classifiers and boosting techniques have been evaluated [17–22]. Some benchmark datasets for BCI design evaluations have been proposed [23] and have met reasonable acceptance.

Accurate assessment of cognitive load from ambulatory electroencephalogram (EEG) could lead to a wide variety of applications for brain interface systems [24]. Of specific interest to us is the concept of augmented cognition (AugCog), which is applicable where the cognitive load of human operators needs to be monitored to design optimal information flow protocols from the computer to the human in order to maximize task performance [25]. These applications include, but are not limited to, vehicle drivers, machinery operators, air traffic controllers, and robotic surgery operators. Optimizing the information flow for seamless human-computer interaction requires the real-time assessments of cognitive states during the execution of certain tasks leading to a prescribed goal. An accurate cognitive load estimator is essential for the successful implementation of assistive systems that are aware of the user's status and environment. Instantaneous estimates of mental state and workload can be used to control the rate and the modality of the information presented to the operator, which in turn helps the operator allocate mental resources to maximize performance [26]. As the envisioned applications require ambulatory EEG recordings, special care must be given to proper signal conditioning, noise and artifact reduction.

The use of EEG, as the basis of assessment in brain-computer interface (BCI) and AugCog systems, is predicated on characteristics such as good temporal resolution, non-invasiveness, low cost, and portability [27]. However, the following factors make it particularly difficult to deal with ambulatory EEG signals: (1) noise resulting from motion artifacts; (2) contamination with muscular activities, including the usual eye movements and blinks; (3) influence of concurrent but irrelevant neural activities; (4) environmental noise; (5) nonstationarity. Under these circumstances, both robustness and precision of the designed system are particularly critical. Furthermore, the system must be portable and able to work in real-time. *The focus of this paper is on feature and channel selection for real-time cognitive state classification based on EEG* in order to address items (1) to (4) in this list. Note that nonstationarity could also be partially addressed to the extent that training session provided sufficiently rich data to represent various sources of nonstationarity.

From a machine learning point-of-view, an EEG characterization system (such as a BCI) requires a robust pattern recognition system to assess the cognitive states or the intent of the operator. A typical classification system contains five parts: preprocessing, feature extraction, dimensionality reduction, classification, and postprocessing. Although any improvement in one of these parts can boost the performance of the system, *in this paper, our focus will be on dimensionality reduction* , because criteria such as accuracy, real-time performance, and wireless networking require all rely on a set of compact features. Furthermore, choosing the most informative and stable feature subset can also partly solve the subject-to-subject transfer, session to session transfer, and nonstationarity problem. The other modules of the classification system were designed following well-established techniques. For example, we employed a standard adaptive filtering technique for the removal of eye artifacts. We used FFT based power spectrum density (PSD) estimation procedures to estimate the power at various frequency bands broadly accepted to be associated with cognitive activity—these estimates served as the primary features for classification. Additionally, we used Gaussian mixtures model (GMM), K nearest neighbor (KNN), and Parzen window density estimate (Parzen) methods for classification. The PSD features constitute a high-dimensional vector that contains information pertinent to the classification of cognitive states, as well as irrelevant components and noise. Direct classification using such input features is undesirable since the unwanted components have an adverse effect on the overall classification performance and the generalization ability of the system. Consequently, a practical technique for extracting the relevant information from these features is necessary.

We present the following: (1) a nonparametric sample estimator for mutual information that combines fast linear ICA solutions with sample-spacing entropy estimators to achieve computational simplicity; (2) EEG channel selection and linear feature projection techniques based on mutual information to achieve dimensionality reduction for computational and generalization benefits.

## 2. METHODS

**Hardware platform**

A mobile wireless sensor suite was assembled using a variety of off-the-shelf components. EEG was collected from 32 channels using a BioSemi Active Two system [28]. Vertical and horizontal eye movements and blinks are recorded with electrodes below and lateral to the left eye. This system integrates an amplifier with an Ag−AgCl electrode—this affords extremely low noise measurements without any skin preparation. Information from the sensors is transmitted (via a combination of Bluetooth, serial port, and USB) to and recorded on a body-worn laptop (Pentium 4.3GHz with 1GB RAM). A base station computer controls the experiment and communicates with the laptop via an 802.11 wireless network.^1^

**Signal processing and classification**

**Figure 1:**
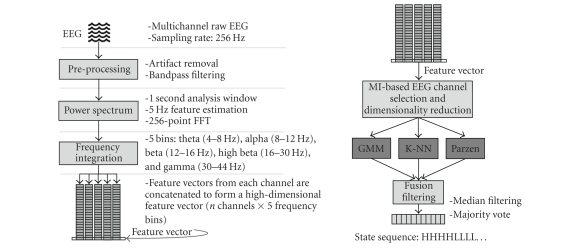
PSD-based feature extraction (left) and dimensionality reduction, classification, and postprocessing flow diagrams (right).

All channels reference the right mastoid. EEG is recorded at 256 Hz sampling frequency while the subject is performing tasks with various cognitive loads. EEG signals are preprocessed to remove eye blinks using an adaptive linear filter based on the Widrow-Hoff training rule [18]. Information from the VEOGLB ocular reference channel was used as the noise reference source for the adaptive ocular filter. DC drifts were removed using high-pass filters (0.5Hz cut-off). A bandpass filter (between 2 Hz and 50 Hz) was also employed, as this interval is generally associated with cognitive activity. The PSD of the EEG signals, estimated using the Welch method [29] with 1-second windows, is integrated over 5 frequency bands: 4–8 Hz (theta), 8–12 Hz (alpha), 12–16 Hz (low beta), 16–30 Hz (high beta), 30–44 Hz (gamma). The energy levels in these bands sampled every 0.2 seconds (i.e., sliding windows with 80% overlap) are used as the basic input features for cognitive classification. The particular selection of the frequency bands is based on well-established interpretations of EEG signals in prior experimental and clinical contexts [24]. The overall schematic diagram of the signal processing system is shown in Figure 1.

In the design phase, the PSD features are used to rank and select EEG channels to reduce dimensionality. For this purpose, we assume that training patterns are representative of the spectral patterns one would expect in the performance environment. The final feature vector, with a much lower dimensionality than the original input, is then fed to a committee of three classifiers. Since the distribution of the feature vectors is unknown, we used both parametric and nonparametric classifiers in the committee: GMM, KNN, and Parzen. The classification component signal flow is illustrated in Figure 1. The GMM is a parametric approach where the class probability distributions are approximated by a small number of Gaussians. KNN is a nonparametric approach where the classification is based on the count of nearest neighbors from each class (can be understood as a variable-size rectangular Parzen estimate of the class distributions). The *Parzen classifier* is a nonparametric approach to estimate the posterior probability of a feature vector . Please check. belonging to a given class, using Gaussian kernels in this case. The estimate is a mixture-of-Gaussians with smooth contributions from all samples and this represents a compromise between discrete votes from nearest neighbors and the small number of Gaussian components of the parametric model. The details of the classifiers are discussed in the appendix. We now describe the EEG channel selection and feature projection procedures in more detail, as this is the main focus of this paper.

## 3. DIMENSIONALITY REDUCTION

Feature extraction is the process of discovering a statistical pattern that can differentiate various classes that lead to distinct observations. In contrast, dimensionality reduction is a process of finding optimal feature vectors with reduced dimensionality from a large pool of candidates to keep the useful information and eliminate irrelevant information. This reduces the computational load and increases the robustness of the classification system. Both feature extraction and dimensionality reduction are important steps in classifying EEG signals. Note that some researchers use the term *feature extraction* to mean dimensionality reduction via linear or nonlinear projections. In our terminology, feature extraction is the process of determining candidate features from raw measurements (in this particular case, the act of calculating energies in five frequency bands from the PSD estimates of all EEG electrodes).

The PSD features of EEG signals constitute a high-dimensional vector (5 frequency bands for 32 EEG channels yield 160 features) that contains information pertinent to the classification of cognitive states, as well as irrelevant components and noise. Direct classification using these raw input features yields poor generalization performance. We therefore propose a mutual information based technique to preserve channels and feature subspaces with maximal generalizable. We, therefore, propose a mutual information based learning technique for finite size training sets to preserve channels and feature subspaces that maximize the generalization of discriminative power. Dimensionality reduction can be achieved by feature transformations. The transformation generates either a new feature space, which is called feature projection; or generates a subset of the original feature space, which is called feature selection. Feature selection is a special case of linear projections where the projection matrix is sparse with only one unit per row. Linear transformations are widely used due to their simplicity and robustness. Therefore, they are often preferred to computationally complex and more fragile nonlinear counterparts, especially with small training sets.

Optimal feature selection coupled with a specific classifier topology, namely the *wrapper* approach, is computationally very complex (combinatorial complexity —overall 2^n^ − 1 feature subsets to evaluate in selection for *n* candidate features); thus, is in feasible for large number of features. In contrast, a filter-based approach, which selects features by optimizing a given criterion, is independent of the classifier and is more flexible, but might not yield classifier-tuned optimal results. Since we use a committee of classifiers, the filter approach is found more suitable.

Principal component analysis (PCA) is a widely used dimensionality reduction technique [30, 31]; however, the projections it finds are not necessarily related to the class labels, hence are not particularly useful in pattern recognition. Linear discriminant analysis (LDA) attempts to eliminate this shortcoming of PCA by finding linear projections that maximize class separability as measured by Fisher's criterion that is based on a unimodal class conditional distribution (e.g., Gaussian) assumption [32]. The LDA projections are optimized based on the means and the covariance matrices of classes, which are not descriptive of an arbitrary multimodal probability density function (pdf). Independent component analysis (ICA) has also been used as a tool to find linear transformations that maximize the statistical independence of random variables [33, 34]. However, like PCA, the projection that ICA finds has no necessary relationship with class labels in itself, hence, are not able to enhance class separability [35].

In the filter approach, it is important to optimize a criterion that is relevant to Bayes risk, which is typically measured by the probability of error (for equal class-error risks). Therefore, a suitable criterion for assessing the *quality* of a low-dimensional feature vector ***f*** (either in selection or projection) is the mutual information (MI) between ***f*** and the class label *c* as defined by

**Figure 2:**
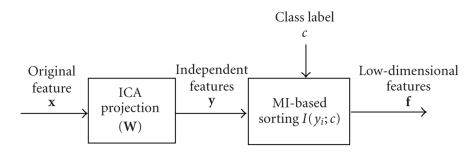
Feature projections using ICA preprocessing and mutual information sorting.

(1) *I_S_*(**f**;*c*) = *H_S_*(**f**)− ∑_c_*p*_*c*_*H_S_*(**f**|*c*)

where *p*_*c*_ is the class prior, *H* *_S_* and *I* *_S_* denote Shannon's definitions of entropy and mutual information [36]. The justification for (1) is intuitively found in argument that **f** should exhibit maximal class label (i.e., cognitive load) relevant information. More formally, lower and upper bounds in information theory that relate mutual information to the Bayes probability of error *p* *_e_* [37, 38], such as *p* *_e_*(**f**) ≤ (*H* *_S_*(*c*) − *I* *_S_*(**f**;*c*))/2 [38], as well as Fano's bound, motivate the use of MI in discriminative dimensionality reduction. Several MI-based methods have been proposed for feature selection [39–43]. However, since features are typically not independent, these approaches cannot guarantee optimal feature selection that would maximize mutual information, and joint information among multiple features (redundancy) is usually ignored or approximated with pairwise mutual information estimates. In this paper, we propose a greedy framework for feature selection and dimensionality reduction based on maximal mutual information as (1) suggests (Figure 2).

### 3.1. Estimating mutual information

A computationally efficient sample estimator for MI that exploits fast linear ICA algorithms to separate mixed features into approximately independent features is proposed. The estimator then employs a one-dimension entropy estimator. In a square invertible ICA transformation ***y*** = ***W*** ^*T*^ **f**, the relationship between the entropy of the low-dimensional features **f** ∈ *ℜ* *^d^* and the entropy of the transformed features *y* satisfies [36]

(2) *H*_*S*_(**f**) = *H*_*S*_(***y***) − log ⁡|**W**|, *H_S_*(**f**|*c*) = *H_S_*(**y** | *c*) − log ⁡|**W***^c^*|

,where **W** is the ICA separation matrix for all data, and **W** *^c^* is the ICA separation matrix for the data from class *c* (in case classes are oriented differently).^2^ If the components of the random vector **y** in (2) are approximately independent, the joint entropy becomes the sum of marginal entropies. Similarly, if **y** conditioned on *c* has approximately independent components, the conditional joint entropy becomes the sum of marginal-conditional entropies:

(3) $$\begin{array}{c} H_{S}(\text{f})=\sum_{l=1}^{d}H_{S}(y_{l})-\log|\text{W}|-I_{S}(\text{y}),\\ H_{S}(\text{f}|c)=\sum_{l=1}^{d}H_{S}(y_{l})-\log|{\text{W}}^{c}|-I_{S}(\text{y}|c). \end{array}$$

Above, *I*_*S*_(***y***) and *I* *_S_*(***y***|*c*) denote any residual mutual information after the linear ICA procedure. Overall, assuming that these residual dependencies are negligible, we have

(4) $$\begin{array}{ll} I_{S}\;(f;c) & =H_{S}\;(f)-\underset{c}{\sum }\;p_{c}\;H_{S}\;(f|c)\\ & \approx \;\overset{d}{\underset{l=1}{\sum }}\;(H_{S}\;(y_{l})\;-\;\underset{c}{\sum }\;p_{c}\;H_{S}\;(y_{l}\;|\;c))\\ & \;-(\log|W|\;-\;\underset{c}{\sum }\;p_{c}\;\;\log|W^{c}|). \end{array}$$

For simplicity, in the following, we further assume that the linear transformations satisfy **W** = **W** *^c^* for all *c*. Thus,

(5) $$I_{S}(\text{f};c)=I_{S}(\text{y};c)\approx \sum_{l=1}^{d}I_{S}(y_{l};c).$$

Consequently, the MI between the classes and *d* -dimensional feature vector can then be computed by evaluating *d* one-dimensional MI estimates as in (5).

**Fast linear ICA solution**

There are several efficient algorithms for solving the linear ICA problem based on a variety of assumptions including maximization of non-Gaussianity, minimization of mutual information, nonstationarity of the sources, and so forth [46–48]. The fourth-order statistical methods can be compactly formulated in the form of a generalized eigendecomposition problem that gives the ICA solution in an analytical form [49]. This formulation will be employed in this work for its simplicity. Under the assumption of iid samples, the separation matrix **W** is the solution to the following generalized eigendecomposition problem:

(6) $${\text{R}}_{\text{f}}\text{W}={\text{Q}}_{\text{f}}\text{W}\Lambda ,$$

where **R**_**f**_ is the covariance matrix of **f** and **Q**_**f**_ is the cumulant matrix estimated using sample averages: **Q**_**f**_​ = ​*E*[**f** *^T^* **f** **f** **f** *^T^*], **R**_f_ tr (**R**_**f**_), *E*[**f** **f** *^T^*]*E*[**f** **f** *^T^*], **R** **f** **R**_f_. Given these matrices, the ICA solution can be easily determined using efficient generalized eigendecomposition algorithms.^3^

Once the ICA transform is determined and employed to obtain ***y*** such that (5) holds (approximately), the marginal mutual information of each independent feature *y* *_i_* with the class label *c* can be computed using (1) and a simple one-dimensional entropy estimator. One needs to estimate the overall feature entropy *H* *_S_*(*y* *_i_*) using all samples regardless of class labels, and the conditional entropy of each class using only the samples from the corresponding class.

**Marginal entropy estimator**

There exist many entropy estimators in the literature for single-dimensional variables [50]. Here, we use sample-spacings estimator, which is based on order statistics. This estimator is selected because of its consistency, rapid asymptotic convergence, and its computational efficiency. Given a set of iid samples {*y*_1_,…,*y*_*N*_} of a random variable *y*, the estimator first sorts the samples in increasing order such that *y*_(1)_ ≤ ⋯ ≤ *y*(*N*). The *m* -spacing entropy estimator is given in terms of the sorted samples by [46]:

(7) H^(y)=1N−m∑i=1N−m log⁡(N+1)(y(i+m)−y(i))m,

where *N* is a sample number. This estimator is based on two assumptions: the true density *p*(*y*) is approximated by a piecewise uniform density determined by *m* -neighbors and outside of the sample range; the contribution of the true density is negligible and/or does not change the expected entropy computed by (7). The selection of the parameter *m* is determined by a bias-variance tradeoff and typically *m* = *N*1/2. In general, for asymptotic consistency, the sequence *m*(*N*) should satisfy

(8) $$\underset{N\to \infty }{\lim}m(N)=\infty \;\;\underset{N\to \infty }{\lim}\frac{m(N)}{N}=0.$$

### 3.2. EEG channel selection using mutual information

In real-time brain interface applications such as the ambulatory cognitive load estimation problem considered in this work, the reduction in the number of input features is further motivated by the limited data acquisition and processing capabilities of the hardware. While collecting measurements from all EEG channels and then projecting their combined feature vector to a lower-dimensional linear or nonlinear manifold would be desirable, the hardware limitations and the prohibitive cost of collecting and processing each additional EEG channel signal beyond the capacity of the hardware imposes us to focus on identifying the salient EEG channels that contain the most useful information for accurate estimation of the cognitive state in the design phase. Each channel yields several (five in our case) features and our goal is to find a quasi-optimal subset of EEG channels such that the MI between features obtained from the selected channels and class labels is maximized for the given number of channels (our hardware can handle up to 7 channels):

(9) $$\underset{\left\{i_{1},\ldots ,i_{m}\right\}}{\max}I_{S}\left(f^{i_{1}},\ldots ,f^{i_{m}};c\right),$$

where **f** *^i^* is the feature vector that contains all features from channel *i*, *c* is the class label, and *m* is the number of EEG channels being considered in **f** ^*T*^ = [**f** ^*i*_1_*T*^, …,**f** ^*i*_m_*T*^]. *I*_*S*_(**f**;*c*) can be estimated using the method described in [Sec subsec3.1]Section .

In order to determine an effective subset of the available features or channels (which encompass multiple features), we rank the channels using a forward incremental strategy. We first select the channel whose features have maximum mutual information with class labels and assign it rank 1. Rank 2 is assigned to the channel that has maximum MI when used in conjunction with the previously selected rank-1 channel. The procedure then ranks iteratively all features or channels taking into account the joint mutual information with previously ranked channels.^4^ Algorithm 1 summarizes the proposed method.

The procedure results in an ordering of EEG channels such that the rank- *d* channel is the optimum choice given the previous *d* -1 channels. While the top *d* channels do not necessarily have to be the best *d* -subset, determining the latter requires a combinatorial search, and is infeasible for very large dimensional situations (such as with 32 EEG channels or 160 features). Using the incremental ranking strategy, the computational complexity is (*n*+1)*n*/2 (*n* is the total number of EEG channels) instead of the (2^*n*^− 1) of exhaustive search. The search procedure could be modified easily to include a channel subtraction phase where a previously ranked channel is removed to the unranked set if it does not contribute to the joint information of the current ranked subset. Another advantage of this method is that, using MI for ranking results in classifier-independent EEG channel ranking, thus it is computationally efficient compared to wrapper techniques (it uses a simple MI estimator and does not require repeated classifier training).

### 3.3. Maximally informative linear feature projections

Even after channel selection, further dimensionality reduction might be desirable to improve classifier generalization performance. This can also be achieved using the maximum MI framework because an invertible transformation does not change the mutual information. In particular, the linear invertible ICA mapping guarantees that *I*_*S*_(**f**;**c**) = *I* *_S_*(*y*;*c*) for *y* = W*^T^* **f**. Furthermore, since (5) holds for the independent features and since MI is a nonnegative quantity, the best *d* -dimensional linear projection consists of the *d* components of *y*, that have maximum individual mutual information with *c*. After the ICA mapping, one needs to evaluate the mutual information *I*_*S*_(*y*_*i*_;*c*) for *i* = 1,…,*n*, *n* is the dimension of the transformed features *y*. The projection matrix then consists of the *d* columns of the ICA matrix *W* that corresponds to the top *d* components of *y*. This projection scheme is illustrated in Figure 2. Typically, the channel selection procedure described in Section 3.2 is employed for selecting the useful sensors motivated by physical constraints; and the feature projection procedure described here is employed to the selected channels to improve classifier robustness and generalization capability in the availability of only a relatively small training data set.

### 3.4. Bias analysis

The approximations in Section 2 introduce an estimation bias to each MI evaluation step. From the derivation, we can see that the bias, defined as the expected difference between the estimation and the true MI, is

(10) E[I^S(f;c) −IS(f;c)]=(log⁡|W|−∑cpclog⁡|Wc|) +(IS(y)−∑cpcIS(y|c)),

where *y* = **W** *^T^* **f** is the ICA transformation.

## 4. EXPERIMENTS AND RESULTS

In this section, we present analyses carried out on data collected from three subjects performing two tasks in multiple sessions (used for training and testing). Note that in many BCI experiments, reports are provided in terms of leave-one-out performance on the complete data set due to scarcity. However, in our experience, this overestimates actual generalization performance (due to nonstationarity being nulled by the leave-one-out procedure).

### 4.1. EEG channel selection

In this experiment, we demonstrate the performance of the channel selection procedure outlined to examine the effectiveness of the selection procedure outlined in Section 3.2. Based on hardware limitations for real-time processing of EEG, the goal of identifying up to 7 channels out of the 30 available ones (we omitted 2 extremely noisy channels in this dataset) is set. Three subjects *S*_1_ – *S*_3_ executed two mental tasks called *Larson* and *n* *-back* [24, 51, 52]. In the Larson task, the subjects are required to maintain a mental count according to the presented configuration of images on the monitor. The combination of mental activities during this task includes *attention*, *encoding*, *rehearsal*, *retrieval*, and *match*. The complexity of this task was manipulated by varying the interstimulus interval (low and high). In the *n* -back task, subjects are required to match the letter in either spatial location or verbal identity in the previous trials. The easy task only requires comparing the current stimuli with the first one, involving the combination of mental activities include attention and match. The difficult task requires comparing the current stimuli with stimuli presented two trials previously, and involves a complex combination of mental activities that includes attention, encoding, rehearsal, retrieval, and match. All three subjects performed both tasks at the two designated difficulty levels. Each case consists of about 3000 data samples in a 150-dimensional feature space (30 EEG channels × 5 frequency bands) with two classes: low and high workloads. We applied the EEG channel-ranking algorithm to the data to study the subject and task dependency of the selected channels. Prior work suggested that the optimal EEG channels may vary for different mental tasks and different subjects.

We first applied the approach on individual subject-task combinations, and obtained specialized EEG channel rankings, designated as *Local* *n* (*n* is the number of the selected EEG channels). To examine the ability to select optimal channels for all tasks and all subjects, we also used data from all subjects and tasks to get another ranking called *Global* *n*. An instance of Local 10 (optimal for subject-task pairs) and Global 10 (optimal across subject-task pairs) EEG channels are shown in Table 1. The 7 channels selected based on literature suggestions for these tasks (see Section [Sec subsec4.1]) are also listed for reference as *Phy 7*. Note that the individual best channels vary for each subject and task combination as expected. Nevertheless, the global ranking strongly coincides with these individual rankings as observed from Table 1.

To validate the proposed method, we employed a committee of 3 classifiers: GMM, KNN, and Parzen, with majority vote and decision fusion on the selected EEG channels. For jackknife evaluation of performance, the data for each case is partitioned to five sets and each set is saved for testing using the other four for training. The confusion matrices are estimated and the correct classification rates are calculated. The classification accuracies averaged over the five test sets are shown in Table 2. Note that the MI-selected channels significantly outperform the literature-motivated channels. On average, keeping 7 or 10 channels does not make significant difference in accuracy. The MI-selected features perform around 80% accuracy on average for all subjects; the specific subject-task optimal selections (local) are observed to be similar to the global selections. This indicates that the proposed channel selection method can partly solve the subject-to-subject transfer and the session-to-session transfer problems.

**Figure 3:**
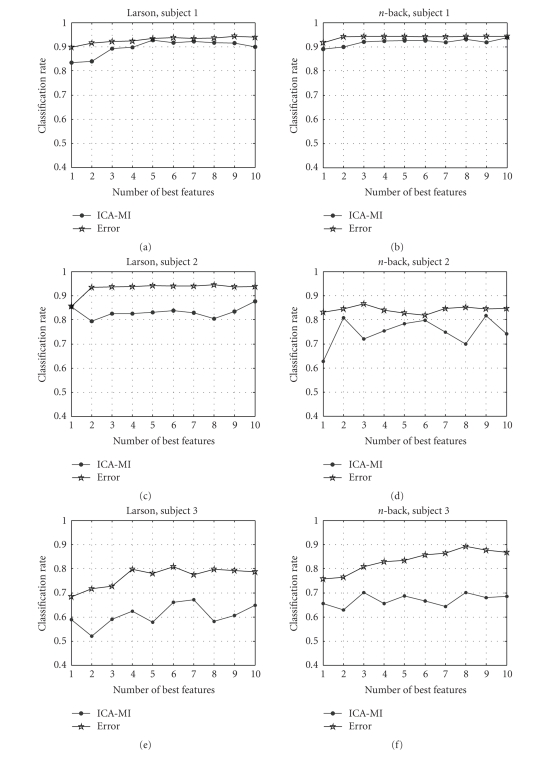
Correct classification rate versus number of optimally selected channels (up to 10, using ICA-MI and error based methods) for three subjects performing two mental tasks.

To provide a wrapper-benchmark for the proposed ICA-MI channel selection method, we also apply error-based ranking to the ICA projections on the same EEG datasets. The error based ranking method uses the same forward search strategy described in the algorithm of [Sec subsec3.2]Section . The difference is, this method uses the classification error of the committee-classifier as its ranking criterion instead of mutual information. The classification results using different channel ranking methods for different subjects and mental tasks are shown in Figure 3 (we only show the classification results for top 10 EEG channels). Horizontal axis denotes the number of selected features used for classification; vertical axis denotes the classification accuracy in percentage. The error based ranking yields more accurate ranking than ICA-MI method. However, it is not practical because it is very slow and inflexible (classifier specific).

### 4.2. Feature projections

In this section, we demonstrate how an optimal ICA-feature . Please check. subspace selected according to the mutual information criterion performs in reducing feature dimensionality without adversely affecting classification performance. Data was collected from one subject as four predetermined ambulatory tasks were executed: *slow walking*, *navigating and counting, communicating with radio*, and *studying written information while standing* . Tasks are assigned class labels from 1 to 4, corresponding to the assigned task. After preprocessing and feature extraction, approximately 6000 data samples were obtained, each with 35-dimensional feature vectors (7 EEG channels with 5 frequency bands each) and a desired class label. In this experiment, the channels corresponded to sites CZ, P3, P4, PZ, O2, P04, F7. These were selected based on a saliency analysis of EEG collected from various subjects performing cognitive test battery tasks [53]. A randomly selected one third of these samples were used as the training set for feature projection and classification, and the remaining two-thirds were used as the test set. The feature projections were obtained as described in [Sec subsec3.3]Section . Correct classification rates for different dimensionality of optimally selected features were evaluated using the classifier committee over 50 Monte Carlo runs (random partitions of training and testing data). To provide benchmarks forthe proposed ICA-MI linear projections, we also present results using other linear feature projection methods. These are ICA transformation followed by classification error based selection (instead of MI), as a wrapper benchmark, and LDA (major generalized eigenvectors of between and within class scatter matrices), as a filter-type common contender. To compare these methods fairly, we normalize the data before we apply the KNN classifier to the projected features (see Appendix 7).

**Figure 4:**
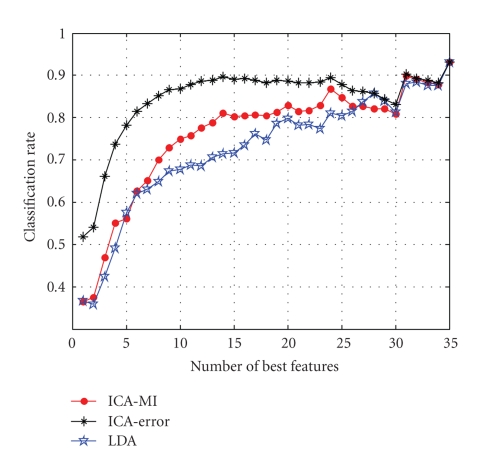
Correct classification rate versus dimensionality of optimally selected features for different methods.

The classification results for different feature ranking methods are shown in Figure 4. The horizontal axis denotes the number of selected features used for classification; the vertical axis denotes the classification accuracy. From Figure 4 we see that ICA-MI can yield an accuracy of 80% with 14-dimensional projections, while the remaining 21 dimensions do not significantly contribute to the classification accuracy. The classification results based on 10, 14, and 35-dimensional optimally selected features using ICA-MI algorithm are compared in Table 3 via the confusion matrix of the classification results (The *i* *j*th entry of confusion matrix **P** shows *P*(decide class *i*| true class is *j*)). Although in this particular experiment keeping all 35 features yielded the best performance, the classification results illustrated here shows that this feature selection method is able to capture the low-dimensional relevant components in the original feature space. This suggests that the additional features may introduce irrelevant and confusing information that might impair the classification accuracy. In conclusion, mutual information based feature projections are expected to eliminate unnecessary dimensions from the feature vector if not improve performance.

The classification result for ICA-error ranking expectedlyexhibits better performance than that of ICA-MI, however, it takes much longer time.^5^ The result of LDA ranking is similar to that of ICA-MI for the first 5 features, but the classification performance decreases dramatically when the number of features increases due to the unimodality assumption. In experiments not shown here, we also compare the proposed feature projection method to the Mermaid-SIG algorithm [54]. The results show that the classification performances are similar. However, the ICA transformation followed by MI sorting algorithm is much faster.

## 5. DISCUSSION

We described a framework based on mutual information maximization to solve the EEG feature/channel selection and dimensionality reduction problems in order to perform cognitive state classification. The initial real-time and offline experiments suggest that the developed practical and fast algorithm that combines ICA transformations and sample-spacing entropy estimators can classify a small set of discrete cognitive states with a reasonable accuracy when combined with 3 parametric and nonparametric classifiers

The experiments demonstrated that the important EEG sites are consistent with prior physiological knowledge —frontal sites associated with working memory tasks are rated high [24]. Some classification performance when using the EEG channels, which were selected from ICA-MI method are even better than the performance of using pre-defined EEG channels. The actual ranking of the most salient sites are highly dependent on subjects and particular tasks they are performing. Nevertheless, a global ranking of EEG sites using the MI principle resulted in virtually no performance loss in classification accuracy on average (across subjects and tasks). This is an important observation that needs to be validated by other BCI researchers, since it indicates that subject-to-subject and task-to-task transfer might indeed be possible, thus making predesigned BCI systems practical.

As a comparison, we also implemented the wrapper approach for feature/channel selection: use classification error as the criterion. As expected, the wrapper approach exhibited better performance than filter approach because it is optimal to specific classifiers; however, it is much more slower, which makes it infeasible in practice with dense array EEG systems that are becoming increasingly popular in BCI research.^6^ The proposed system is feasible; however, the nonstationarity of the EEG data still poses a great challenge making session-to-session transfer a difficult problem to solve. This means we have to retrain the system for different subjects and different sessions, unless a very large training set encompassing a variety of operating conditions, numerous subjects, and tasks is available. We have utilized PSD-based features, and perhaps higher-order statistics or wavelet-based time-frequency features are more stationary and could lead to more robust designs. Future work will focus on determining *better* features.

## Figures and Tables

**Algorithm 1:**
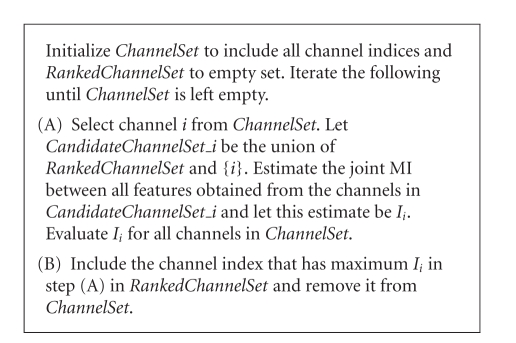


**Table 1:** Optimal EEG channels illustration. Phy 7: 7 EEG channels from physiological literature; Local 10: 10 best EEG channels evaluated from individual subject-task pair; Global 10: 10 best EEG channels evaluated from pairs (boldface highlighted).

Phy 7			Cz, P3, P4, Pz, **O2** , PO4, **F7**
Local 10	S_1_	Larson	CP5, **Fp2** , **FC5** , Fp1, C4, P4, **F7** , **AF3** , P7, **FC6**
		*n* -back	**AF3** , **FC5** , Fp1, **Fp2** , **F8** , **F7** , **FC6** , **O1** , **CP6** , P4

	S_2_	Larson	**Fp2** , **O1** , AF4, **F7** , C3, PO3, **FC6** , CP2, C4, Pz
		*n* -back	C4, **O1** , **F8** , Fz, **F3** , **FC5** , FC1, C3, Cz, CP1

	S_3_	Larson	**Fp2**, **F8**, **F7**, **FC5**, **FC6**, **AF3**, C3, F4, P4, AF4
		*n* -back	CP5, **F8** , C4, **FC6** , **Fp2** , **FC5** , P3, AF4, C3, P7

Global 10			Fp2, FC5, O1, F3, FC6, F8, F7, AF3, O2, CP6

**Table 2:** Correct classification rate for three subjects: S , S , and S , in two mental tasks: Larson and *n* -back, for different subsets of EEG channels. Average is arithmetic average of the 6 correct classification rates for a particular EEG channel subset.

		Phy 7	7 Local	10 Local	7 Global	10 Global
S_1_	Larson	0.78	0.92	0.90	0.92	0.85
	*n* -back	0.86	0.92	0.94	0.93	0.92

S_2_	Larson	0.76	0.83	0.88	0.83	0.87
	*n* -back	0.56	0.75	0.74	0.79	0.73

S_3_	Larson	0.53	0.67	0.65	0.59	0.65
	*n* -back	0.54	0.64	0.68	0.74	0.72

Average		0.67	0.79	0.80	0.80	0.79

**Table 3:** Confusion matrix for classifiers on 4 cognitive states using 10, 14, and 35-dimensional input feature vectors.

Dimensions	10-dimensional input	14-dimensional input	35-dimensional input
Confusion matrix	$\left[\begin{array}{cccc} 0.38 & 0.33 & 0.25 & 0.04\\ 0.03 & 0.82 & 0.15 & 0\\ 0 & 0 & 1 & 0\\ 0 & 0.01 & 0.24 & 0.75 \end{array}\right]$	$\left[\begin{array}{cccc} 0.6 & 0.22 & 0.17 & 0.01\\ 0.01 & 0.91 & 0.08 & 0\\ 0 & 0 & 1 & 0\\ 0 & 0.01 & 0.18 & 0.82 \end{array}\right]$	$\left[\begin{array}{cccc} 0.6 & 0.29 & 0.1 & 0.01\\ 0.02 & 0.83 & 0.15 & 0\\ 0 & 0 & 1 & 0\\ 0 & 0 & 0.02 & 0.98 \end{array}\right]$

## Footnotes

^1^ A real-time AugCog system based on the selected channels is implemented successfully in a communication-routing system that prioritizes information and messages for timely delivery to the subjects in a high-communication task, resulting in increased accuracy of situation awareness (measured by correct responses to questions in postsession interview). Besides EEG, the system incorporates a wearable arousal meter. This unit senses a subject's electrocardiogram (ECG) signals and outputs interbeat interval data in conjunction with a derived measure of a subect's cognitive arousal. The details of this implementation and results are not the subject of this paper.

^2^ Given an arbitrary random vector ***f***, one can always find a nonlinear transformation ***y*** = ***g***(***f***) that is invertible and results in independent components ***y*** = {***y***_1_,…,***y*** *_n_*} [44]. However, in small datasets, finding a robust nonlinear ICA solution is difficult. An approximate linear ICA solution can be sufficient [45].

^3^ Note that fourth-order cumulant-based ICA algorithms typically require a much larger sample size than information theoretic methods such as Infomax [49] and Mermaid [50], thus has much larger estimation variance for a given sample size. Also, joint diagonalization of more than two higher-order cumulants is usually preferred.

^4^ Note that when ranking channels, since all features associated with the signals of an EEG channel must be included or excluded simultaneously, the algorithm considers concatenating feature vectors of channels to form candidate feature subsets. In contrast, if all channels could be measured, one could also do feature subset selection using the same algorithm, this time concatenating features individually to form candidate subset feature vectors.

^5^ As an indication of the order-of-magnitudes of difference in speed, in this experiment, it takes a few seconds for the ICA-MI projection, but it takes tens of hours for ICA-error ranking.

^6^ We applied both classification error-based wrapper approach and our MI-based filter approach on the same data set with the same computer platform. The wrapper approach used more than one day. In contrast, the proposed filter approach used only 20 minutes on the same computer using Matlab.

## APPENDIX

### A. Classifiers

Gaussian mixture model (GMM) classifier Gaussian mixture models are widely used to model the probability and B. density functions. In this paper, they are employed to approximate class-conditional distributions. It is assumed that each class distribution consists of four Gaussian models and the parameters of the mixture is optimized using the expectation-maximization (EM) algorithm [55]. The estimated distributions are then utilized to form an approximate Bayes classifier.

K nearest neighbor (KNN) classifier The KNN classification approach is a nonparametric technique that makes no assumptions about the form of the probability densities underlying a particular set of data. Given a particular test sample, the *K* nearest training samples (usually in an Euclidean sense) are determined and the test sample is assigned to the class which lends the most neighbors to this set. It can be shown that if *K* is large, this classifier will approach the best possible classification performance given by the true Bayes classifier [56].

Parzen window classifier Parzen windowing [57] is a nonparametric density estimation technique. It is employed to estimate the class distributions and to form a nonparametric approximation to the Bayes classifier. In this context, it serves as a bridge between the KNN where each sample contributes discretely to the decision (depending on whether they are in the neighborhood or not) and the GMM classifier where each sample indirectly contributes to the Gaussian models. In our implementation, we used Gaussian window functions, thus the Parzen classifiers is essentially a KNN classifier with decreasing influence by distance, and at the same time it is a GMM itself, where a Gaussian is placed on each sample.

Fusion The classifiers output a decision at 10 Hz and the majority vote determines the final cognitive state estimate. The Parzen classifier decision was accepted when there was no agreement. It is also assumed that this state will not change over a period of 2 seconds, thus a median filter applied to the most recent 10 decisions is utilized to smoothen the classification output. This postprocessing step significantly improves performance and reduces flickering.

### B. Scale normalization across linear projections

When comparing different linear projection propositions using a classifier whose training and performance depends on Euclidean sample distances and angles for the purpose of having a controlled environment, it is important to guarantee that the classifier performances are not affected by Euclidean transformations of data across projection methodologies. Data normalization to satisfy this desirable property is essential to conclude with certainty that differences in performances of classifiers due to various linear projections are invariant to affine transformations.

Suppose that a linear projection matrix **W** ∈ *ℛ* ^*m*^ × ^*n*^, where *m*<*n*, is proposed as the *optimal projection* according to the criterion of that particular technique (e.g., PCA, LDA, ICA, MI would yield different propositions). Let **W** = **U** **D** **V** *^T^* be the singular value decomposition of this matrix, where **D** is the diagonal matrix of eigenvalues, and **U** and **V** are orthonormal left and right eigenvector matrices. Define the multiplicative group inverse **W** ^+^ = **V** **D** ^+^ **U** *^T^*, where D^+^_*ii*_ = D^±1^_*ii*_ if **D** *_ii_* ≠ 0 and D_*ii*_^+^ = 0 if **D** *_ii_* = 0 (i.e., **D** ^+^ is the group inverse for diagonal matrices under multiplication).

In the comparison of linear projections using a particular classifier (e.g., KNN, SVM, etc.), instead of utilizing the samples obtained by **y** = **W** **x**, where **y**∈ *ℛ* ^*m*^, utilize the samples generated with **z** = **W** ^+^ **W** **x**. Note that, although **z** ∈ *ℛ* ^*n*^, since rank(**W** ^+^ **W**) = rank(**VI** *_m_* **V** *^T^*) = *m* —where **I** *_m_* = diag(1,⁢,1,0,⁢,0) is *n* × *n* diagonal with *m* ones on its diagonal —the samples of the random vector **z** lie on an *m* -dimensional hyperplane determined by the rows of **W**. The variable **z** is a scale-normalized version of the desired projection**y**, and its use eliminates the problems that might arise from the scale dependency of particular classifier topologies and improper training procedures that might not take these into account.
